# Continuing a scientific dialogue between sectors on health and economics

**DOI:** 10.2471/BLT.24.291722

**Published:** 2024-05-01

**Authors:** Ritu Sadana, Rajat Khosla, Rachel Gisselquist, Kunal Sen

**Affiliations:** a Secretariat, WHO Council on the Economics of Health for All, World Health Organization, Avenue Appia 20, 1211 Geneva 27, Switzerland.; b United Nations University International Institute for Global Health, Kuala Lumpur, Malaysia.; c United Nations University World Institute for Development Economics Research, Helsinki, Finland.

The World Health Organization (WHO) Council on the Economics of Health for All offers a powerful narrative on the relationship between economies and health. The council argues that to build an economy that guarantees health for all, a whole-of-society approach is required to take advantage of all policy levers. The council concludes that health is not only a by-product of economic growth nor just one sector, but a cross-cutting lens through which to view every sector.^1^ Long-term policies and actions must therefore adopt this broadened framing of health to reshape and redirect the economy, for the good of all people and the planet.^2^

This issue of the *Bulletin of the World Health Organization* includes papers that highlight diverse global and national approaches, address the work, themes and recommendations of the council,^3^ and offer promising solutions.

The council calls for rethinking values to redirect investments to deliver the goal of health for all. In this theme issue, a research group identifies that 140 of 194 countries outline a constitutional right to health, and another 37 recognize health-care entitlements through legislation or judicial means; its authors argue that stronger health governance and greater investments in health are needed to enforce rights.^4^ In addressing the root causes of health and well-being, another study suggests that meaningful advancements in mental health necessitate addressing sources of shared distress and promoting mental health as part of a well-being economy.^5^ Challenging how we understand progress, a paper proposes the inclusion of population health within a revised System of National Accounts – the basis of calculating gross domestic product.^6^

To increase the level of financing for health and the quality of finance, developing a health taxonomy to guide sustainable investment decisions through engaging public and private sectors is proposed.^7^ Given the limited financing for pandemic preparedness and response measures, a review identifies gaps that impede accurate quantification of shortfalls and evaluations needed before developing more robust global financing instruments.^8^

The council recognizes the interlinked nature of health, inequality and climate crises, and calls for investments that tackle these aspects together. A study offers a forward-thinking example, proposing that breastfeeding be recognized in food and well-being statistics, and investments in breastfeeding be considered a carbon offset in global financing arrangements for sustainable food, health and economic systems.^9^ Another paper provides an overview of climate finance mechanisms and discusses how the health sector can leverage these funds for climate-resilient health systems.^10^ As the health sector contributes approximately 5% to greenhouse gas emissions, a study discusses how health financing policies can be designed to aid mitigation measures and prepare for the impact of climate change on health.^11^

As advocated by the council, innovation for health for all requires challenging the prevailing notion of private sector-led and market-driven practices as the only way forward. A paper does so by examining the mRNA [messenger ribonucleic acid] Vaccine Technology Transfer Programme as a promising initiative to develop an innovation system and reshape the global vaccine market.^12^ Given the global asymmetries in access, another paper describes the Brazilian government’s value-driven industrial policy that has attracted public and private investments and increased equitable access to essential health products.^13^ An article describes the Group of 20 Joint Finance and Health Task Force as a mechanism to improve global coordination in pandemic preparedness and response, and to optimize financing mechanisms.^14^

The council recognizes the need to strengthen public sector capacity to drive transformative change. Addressing the differing goals of human health, animal husbandry, trade, agriculture and environment sectors, a paper outlines steps to increase whole-of-government financing of National Action Plans addressing antimicrobial resistance, given that 89% of such plans across 177 countries with a plan remain unfinanced.^15^ A study discusses that the challenge for most member countries in the Organisation for Economic Co-operation and Development is to find a fiscally sustainable path to fund increases in health investments needed to secure equitable health outcomes; its Joint Network of Senior Budget and Health Officials from finance and health ministries facilitates dialogue and discussion of policy options.^16^

Building an economy for health and well-being requires ongoing dialogue engaging all sectors, with participatory approaches^17^ and practical tools.^18^ This theme issue will be discussed at events organized by the Finnish government and WHO during the World Health Assembly in 2024, during which a resolution on economics of health for all^19^ will be considered.
